# The impact of cardiac rehabilitation on atrial fibrillation recurrence after pulmonary vein isolation: results of a large retrospective study

**DOI:** 10.3389/fcvm.2025.1717749

**Published:** 2026-01-27

**Authors:** Rana Önder, Gitte Geebelen, Martijn Scherrenberg, Paul Dendale, Lien Desteghe, Hein Heidbuchel, Johan Vijgen

**Affiliations:** 1 Faculty of Medicine and Life Sciences, Hasselt University, Hasselt, Belgium; 2 Heart Center Hasselt, Jessa Hospital, Hasselt, Belgium; 3 Research Group Cardiovascular Diseases, GENCOR, University of Antwerp, Antwerp, Belgium; 4 Department of Cardiology, Antwerp University Hospital, Edegem, Belgium; 5 Department of Nursing and Midwifery Sciences, Centre for Research and Innovation in Care (CRIC), University of Antwerp, Antwerp, Belgium

**Keywords:** atrial fibrillation, cardiac rehabilitation, pulmonary vein isolation ablation, recurrence, retrospective study

## Abstract

**Aims:**

Pulmonary vein isolation (PVI) is a common treatment for atrial fibrillation (AF), but many patients experience recurrences. Physical activity can be beneficial in reducing recurrences. In Belgium, patients can join a reimbursed cardiac rehabilitation (CR) program after PVI, although not all do. This study retrospectively examined the impact of CR on AF recurrence after PVI.

**Methods:**

A database of AF patients who underwent primary PVI ablation between 2007 and 2020 was analysed for documented AF recurrences. Patients were retrospectively divided into control and intervention groups based on their participation in the CR program.

**Results:**

Of 1,765 included patients, 1,177 were controls, and 588 participated in CR [median age 64 (56–70) years (*p* = 0.186), BMI 27.7 kg/m^2^ (*p* < 0.001), median follow-up 1,516 days (*p* < 0.001)]. The prevalence of hyperlipidemia (*p* = 0.009), smoking history (*p* = 0.001), and sleep apnea (*p* = 0.009) differed between both groups. Survival analysis showed no significant difference overall (*p* = 0.340), although there was an intriguing crossover of the recurrence curves after about 1,500 days. Despite an initial higher recurrence, patients who followed CR had a 32.2% lower odds of AF recurrence from 1 year post-PVI until study end (OR: 0.677 *p* = 0.005). After 3 months, BMI decreased significantly in the intervention group and increased in the control group (between-group *p* = 0.004).

**Conclusion:**

Overall, we did not observe a lower AF recurrence in post-PVI patients with a CR program. Nevertheless, physical activity from the CR program may have long-term benefits based on weight loss and VO_2_ max increase.

## Graphical Abstract

**Figure:**
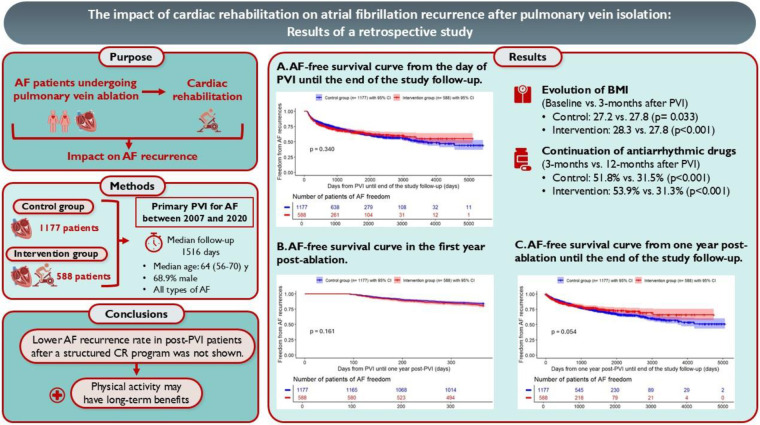

## Summary

This study examined how cardiac rehabilitation (CR) affects the chances of atrial fibrillation (AF) recurrences after a procedure called pulmonary vein isolation ablation.

- Rehabilitation patients did not have fewer AF episodes over the full study period.
- However, physical activity from the CR program may have long-term advantages based on the proven weight loss and increase in VO_2_ max, since the curves significantly diverged after 1 year, showing lower AF recurrence rate in rehabilitation patients.

## Introduction

Atrial fibrillation (AF) is associated with high morbidity and mortality due to an increased risk for heart failure (HF), embolism and/or stroke (1). Several risk factors are related to the development and progression of AF, like overweight, physical inactivity, hypertension, sleep apnea, hyperlipidemia, diabetes, and alcohol consumption (1). Although many patients undergo pulmonary vein isolation (PVI) as the main treatment option, there is a recurrence of AF in 20%–30% after PVI (2). In Belgium, patients are offered a reimbursed cardiac rehabilitation (CR) program after their PVI procedure. This program mainly consists of physical exercise sessions, and optional sessions focusing on implementing lifestyle changes can be added.

Previous studies have shown that CR is beneficial (i.e., it reduces cardiovascular mortality and hospital admissions and improves quality of life) for various heart conditions, such as HF and coronary heart disease (3, 4). However, there is a lack of evidence that such (standardised) CR programs for AF patients after ablation can positively impact rhythm control. The limited evidence may contribute to the low participation rate of AF patients in CR programs. Despite limited research in an AF population, a study by Ritchey et al. indicated that less than 25% of eligible AF patients participated in the CR program (5). Nevertheless, several studies, mainly by the research group of Sanders et al., have demonstrated that physical exercise and addressing risk factors are crucial elements of AF treatment (6–11). Their risk factor management consisted of a dedicated clinic focusing on weight loss (i.e., a 10% reduction) and exercise programs. Patients in this program only visited the clinic every 3 months, according to American College of Cardiology guidelines. The general conclusion of these trials is that the applied risk factor management is associated with a reduction in AF burden (6–11). However, these risk factor management programs are different from the CR program in Belgium, where the main focus is on two or three exercise sessions of 1 h per week to improve cardiovascular fitness.

Therefore, this Belgian retrospective study aimed to investigate the impact of CR on AF recurrence after PVI in a large dataset.

## Methods

### Study design and study population

This retrospective, single-centre, non-randomised cohort study was performed at the Jessa Hospital in Hasselt, Belgium. An extensive database was compiled with data of AF patients (age ≥ 18 years) who had a primary PVI ablation between 01/01/2007 and 31/12/2020. Primary PVI ablation was defined as the first PVI ablation performed to treat AF or AF in combination with atrial flutter. Exclusion criteria were patients with current excessive thyroid dysfunction or with a history of thyroid dysfunction (including amiodarone-induced), pregnancy or breastfeeding, and intensive exercise or sports (>1 h a day or >7 h a week) prior to AF ablation. All patients receiving an ablation were given the option to participate in a reimbursed CR program. For this study, the patients were retrospectively divided into two groups. The intervention group consists of patients who participated in the standardised CR program in addition to standard care. The control group only received standard care, which implies a cardiology follow-up 3 and 12 months after the ablation. This study was approved by the Ethical Committee of Hasselt University (UHasselt) and Jessa Hospital Hasselt (approval number: 16.46/cardio16.08), and it was registered at ClinicalTrials.gov (NCT03389633).

### Study procedure and data collection

The medical record of the patients was used to collect all demographic parameters and medical history (e.g., risk factors and comorbidities, echocardiographic parameters, lab parameters, AF-related parameters, CR-related parameters, and medication) (Supplementary Table S1). The date of the first PVI ablation was defined as the baseline for each patient, and all patients had a follow-up of at least 1 year and had a common end of study follow-up, which was 31.12.2021.

The standard CR program starts as soon as possible after the ablation, usually within 1–3 weeks after PVI. The program consists of 45 exercise sessions focused on physical exercise and lifestyle modification advice. Each patient follows an individualized training program under the supervision of physiotherapists, consisting of two to three exercise sessions of 1 h per week. The individualized program is based upon the results of an initial ergospirometry (i.e., the maximum oxygen uptake capacity (VO_2_ max in mL/kg/min) and the maximum power output (power max in Watt), as well as the first (VT1) and second (VT2) ventilatory thresholds) and shared decision making in consultation with the patient. The training devices are set up based on the VT1, and patients with AF should not exercise at levels higher than VT2. This exercise program mainly consists of a combination of 15–20 min both on the exercise bike, treadmill, and recumbent cycle. Resistance training is also advised for those who can perform it and patients have the opportunity to participate in information sessions regarding lifestyle changes (e.g., weight loss, healthy diet, stress, quitting smoking). In addition to physical training and education, the CR program provides personal coaching from the dietitian to help in weight reduction.

### Outcomes

The primary outcomes of this study focused on the time between 3 months after the first PVI ablation and the first documented AF recurrence (with a common end of the study follow-up, i.e., December 2021). AF recurrence means a documented AF episode or a redo PVI. Documented AF can be an episode (longer than 30 s) detected by a Holter or ECG documented in the medical record. This database did not include the AF episodes that occurred within the 3 months after the PVI procedure (i.e., the blanking period).

Secondary outcomes were the documentation and the time of the first AF recurrence between 3 months post-PVI until 1-year follow-up, and the time of the first AF recurrence from 1-year post-PVI until the end of the study follow-up. Other secondary outcomes were the factors associated with AF recurrences during the study follow-up, the total number of AF recurrences between the 3-month blanking period and 1 year after the first PVI ablation. Tertiary outcomes included the evolution of patients’ body mass index (BMI), the treatment with antiarrhythmic drugs, and the change in maximal oxygen uptake (VO_2_ max) and the maximum power output.

### Statistical analyses

The study population consisted of all PVI ablations performed at Jessa Hospital between January 2007 and December 2020. All data collected from the medical records was analysed using SPSS 29.0, RStudio 2023.12.1, or SAS 9.4. The Shapiro–Wilk test was used to check the normality of the data. All continuous data were non-normally distributed data and reported as median and interquartile range (IQR). Differences between the two groups were compared using the Mann–Whitney *U* test, and the Chi-square test. The primary and secondary outcomes (i.e., time to recurrence and event-free survival curves) were tested using a Kaplan–Meier survival analysis (RStudio), whereas the documentation of AF recurrence was analysed with the Chi-square test. Given the violation of the proportional hazards assumption, the two survival curves were compared using a combination test consisting of a set of weighted log-rank statistics. The test was implemented using the Karrison weighting scheme, which provides sensitivity to differences in hazard that may occur early, late, or throughout the follow-up period in the presence of non-proportionality (12). Logistic regression was performed to evaluate the predictors associated with AF recurrences, and it was reported as odds ratios (OR) with 95% confidence intervals (CI). The logistic regression models included variables to correct for selection bias of treatment and potential confounding. The following characteristics were included in the models: age, gender, hypertension, diabetes, heart failure, sleep apnea, valvular heart disease, coronary artery disease, and COPD/asthma. In order to determine the association of other variables collected with AF recurrences, a stepwise variable selection procedure was carried using the variables reported in Supplementary Table S2. The significance level for a variable to enter the model was set at 0.25, and the significance level for a variable to remain in the model was set at 0.05. The characteristics used in the covariate adjustment, were not considered in the variable selection procedure and remained in the model. On the other hand, one-to-one propensity-score matching was performed, however this substantially reduced the sample size. To avoid loss of information, inverse probability of treatment weighting (IPTW) was subsequently applied using the same covariates as included in the logistic regression model. Given (1) the loss of sample size with matching, (2) evidence that covariate adjustment performs similarly to IPTW, (3) the lack of methods for incorporation of propensity-score weights in test that compare two survival curves when non-proportional hazards are present, the approach of covariate adjustment was ultimately retained as our main analysis. The differences in the evolution of BMI and the treatment with antiarrhythmics between both groups were analysed using the Wilcoxon signed-rank test and the McNemar test. Linear mixed model analysis was used to evaluate the between-group differences over time for BMI and a generalized mixed model was used to analyse the between-group differences over time for treatment with antiarrhythmics. Results with a *p*-value < 0.05 were accepted as statistically significant.

## Results

### Patient characteristics

**Figure 1:**
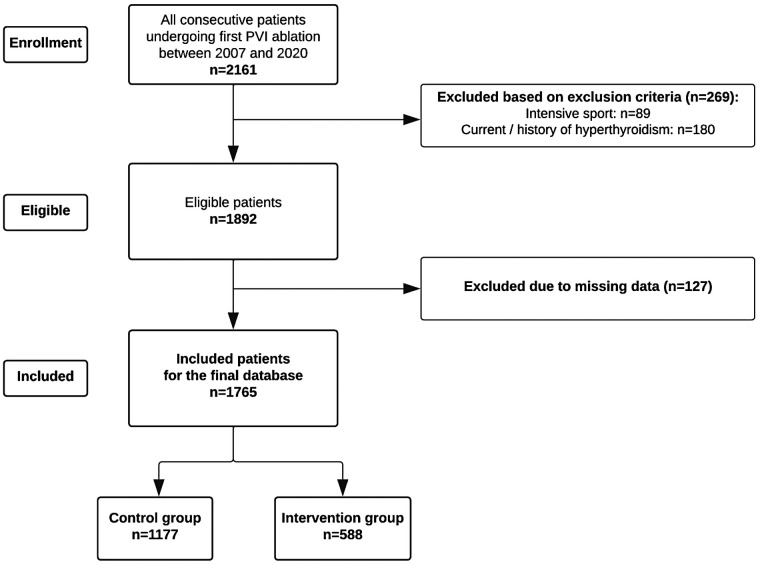
Flowchart of the screened and included patients. PVI, pulmonary vein isolation.

In total, 2,161 patients underwent a first PVI ablation between January 2007 and December 2020. Of those, 269 patients (12.4%) were excluded (Figure 1). A total of 1,765 patients were included in the final database, of which 1,177 (66.7%) patients who did not follow the CR program served as controls, and 588 (33.3%) patients followed the CR program (intervention group). Patients had a median follow-up of 1,516 days (900–2,417), which was significantly different in the control and intervention groups (median of 1,667 vs. 1,232 days; *p* < 0.001). Table 1 shows the demographic characteristics. The median age of all 1,765 included AF patients was 64.0 (56.0–70.0) years, of which 68.9% were male. At baseline, various patient characteristics were comparable between the control and intervention groups, i.e., the median age (63.0 vs. 64.0 year respectively; *p* = 0.186), male gender [*n* = 825 (70.1%) vs. *n* = 391 (66.5%); *p* = 0.121], and the percentage with paroxysmal AF [*n* = 830 (70.5%) vs. *n* = 391 (66.5%); *p* = 0.165]. On the other hand, there was a significant difference in prevalence of hyperlipidemia before PVI [control group: *n* = 719 (61.1%) vs. intervention group: *n* = 401 (68.2%); *p* = 0.009], sleep apnea [control group: *n* = 80 (6.8%) vs. intervention group: *n* = 61 (10.4%); *p* = 0.009], smoking history [control group: *n* = 479 (40.7%) vs. intervention group: *n* = 289 (49.1%); *p* = 0.001], and having a CVA post-PVI [control group: *n* = 9 (0.8%) vs. intervention group: *n* = 11 (1.9%); *p* = 0.038] between both groups. Remarkably, patients had a significantly different BMI, but we observed a higher BMI in the CR group in comparison to the control patients at baseline (28.3 vs. 27.2 kg/m^2^; *p* < 0.001) and 3-month follow-up visit (27.8 vs. 27.1 kg/m^2^; *p* = 0.008). The type of ablation was significantly different between both groups (*p* = 0.017): the majority underwent radiofrequency ablation [control group: *n* = 768 (65.3%) vs. intervention group: *n* = 343 (58.3%)], followed by laser ablation [control group: *n* = 408 (34.7%) vs. intervention group: *n* = 244 (41.5%)] and electroporation [control group: *n* = 1 (0.1%) vs. intervention group: *n* = 1 (0.2%)].

**Table 1:** Demographic table.

	Total (*n* = 1,765)	Control group (*n* = 1,177)	Intervention group (*n* = 588)	*p* value*
Age, years, median (IQR)	64.0 (56.0–70.0)	63.0 (56.0–70.0)	64.0 (57.0–70.0)	0.186
Male, *n* (%)	1,216 (68.9%)	825 (70.1%)	391 (66.5%)	0.121
Anthropometric measures at baseline				
Length, *m*, median (IQR)^a^	1.74 (1.67–1.80)	1.74 (1.67–1.80)	1.74 (1.66–1.80)	0.227
Weight, kg, median (IQR)^b^	84.0 (74.0–95.0)	83.0 (73.0–94.0)	85.0 (75.0–97.0)	**0** **.** **026**
BMI, kg/m^2^, median (IQR)^c^	27.7 (24.9–30.8)	27.2 (24.7–30.6)	28.3 (25.6–31.2)	**<0** **.** **001**
Anthropometric measures at month three first follow-up				
Weight, kg, median (IQR)^d^	83.0 (73.0–94.7)	83.0 (73.0–93.6)	84.0 (74.0–96.0)	0.120
BMI, kg/m^2^, median (IQR)^e^	27.5 (24.8–30.7)	27.1 (24.6–30.5)	27.8 (25.2–30.9)	**0** **.** **008**
Risk factors and comorbidities before ablation, *n* (%)				
Family history	845 (47.9%)	554 (47.1%)	291 (49.5%)	0.680
Hypertension	878 (49.7%)	571 (48.5%)	307 (52.2%)	0.148
Diabetes mellitus	158 (9.0%)	109 (9.3%)	49 (8.3%)	0.487
Hyperlipidemia	1,120 (63.5%)	719 (61.1%)	401 (68.2%)	**0** **.** **009**
Smoker	768 (43.5%)	479 (40.7%)	289 (49.1%)	**0** **.** **001**
Coronary artery disease	281 (15.9%)	185 (15.7%)	96 (16.3%)	0.772
Heart failure	263 (14.9%)	162 (13.8%)	101 (17.2%)	0.064
Cardiac surgery	128 (7.3%)	86 (7.3%)	42 (7.1%)	0.904
Asthma or COPD	150 (8.5%)	97 (8.2%)	53 (9.0%)	0.623
Sleep apnea	141 (8.0%)	80 (6.8%)	61 (10.4%)	**0** **.** **009**
Pacemaker	127 (7.2%)	81 (6.9%)	46 (7.8%)	0.510
Artificial valve	23 (1.3%)	17 (1.4%)	6 (1.0%)	0.460
Valvulopathy	33 (1.9%)	23 (2.0%)	10 (1.7%)	0.713
Terminal renal insufficiency	5 (0.3%)	3 (0.3%)	2 (0.3%)	0.750
Risk factors and comorbidities after ablation^i^, *n* (%)				
Smoker	198 (11.2%)	140 (11.9%)	58 (9.9%)	0.179
TIA	23 (1.3%)	14 (1.2%)	9 (1.5%)	0.550
CVA	20 (1.1%)	9 (0.8%)	11 (1.9%)	**0** **.** **038**
Hyperthyroidism	46 (2.6%)	27 (2.3%)	19 (3.2%)	0.243
Hypothyroidism	37 (2.1%)	25 (2.1%)	12 (2.0%)	0.910
Echocardiographic measures				
Ejection fraction, %, median (IQR)^f^	60.0 (55.0–62.0)	60.0 (55.0–62.0)	60.0 (55.0–61.0)	0.958
LA enlargement, >34 mL/m^2^, *n* (%)^g^	1,375 (77.9%)	918 (78.0%)	457 (77.7%)	0.811
Mitral valve regurgitation, *n* (%)^h^				0.402
0/4	304 (17.2%)	210 (17.8%)	94 (16.0%)	
1/4	1,151 (65.2%)	764 (64.9%)	387 (65.8%)	
2/4	230 (13.0%)	153 (13.0%)	77 (13.1%)	
3/4	23 (1.3%)	12 (1.0%)	11 (1.9%)	
4/4	3 (0.2%)	3 (0.3%)	0 (0.0%)	
AF related parameters				
AF type				0.165
Paroxysmal	1,221 (69.2%)	830 (70.5%)	391 (66.5%)	
Persistent	531 (30.1%)	340 (28.9%)	191 (32.5%)	
Permanent	13 (0.7%)	7 (0.6%)	6 (1.0%)	
Type of ablation				**0** **.** **017**
Radiofrequency	1,111 (62.9%)	768 (65.3%)	343 (58.3%)	
Laser	652 (36.9%)	408 (34.7%)	244 (41.5%)	
Electroporation	2 (0.1%)	1 (0.1%)	1 (0.2%)	
Ablation with complication	45 (2.5%)	26 (2.2%)	19 (3.2%)	0.198
Redo ablation	345 (19.5%)	235 (20.0%)	110 (18.7%)	0.535

Baseline characteristics of included AF patients (*n* = 1,765). Non-normally distributed values are demonstrated as median (IQR) and categorical values as *n* (%). BMI, body mass index; COPD, chronic obstructive pulmonary disease; CVA, cerebrovascular accident; LA, left atrium; TIA, transient ischemic attack.

a 13 Unknown.

b 71 Unknown.

c 73 Unknown.

d 161 Unknown.

e 164 Unknown.

f 183 Unknown.

g 18 Unknown.

h 54 Unknown.

i The first documentation after the ablation.

* Comparison between the control group and intervention group (*p* < 0.05).

Bold values are the parameters with a significant *p* value.

### AF-free survival

**Figure 2:**
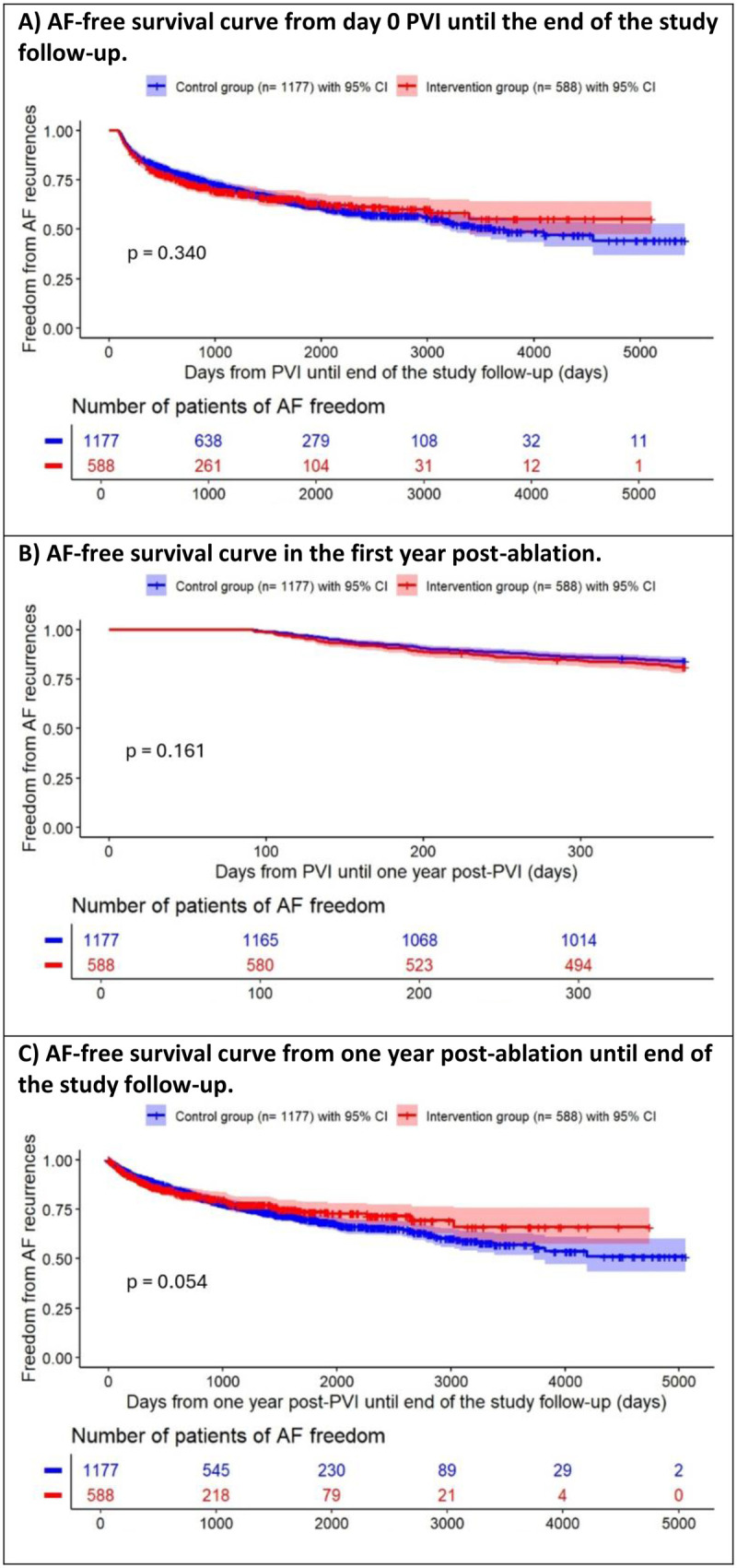
Kaplan–Meier curves for AF-free survival after PVI (with 3 months initial blanking period) **(A)** from day 0 post-ablation until the end of the study follow-up, **(B)** in the first year post-ablation, and **(C)** from 1 year post-ablation until end of the study follow-up. AF, atrial fibrillation; PVI, pulmonary vein isolation. *P*-value is obtained from the combination test.

The proportion of AF recurrence was comparable in the intervention and control groups within 1 year post-ablation (18.9% vs. 15.9%, respectively; *p* = 0.198). Figure 2A demonstrates the AF-free survival (from PVI until the end of study follow-up) of patients after their primary PVI procedure, with no significant difference between both groups (*p* = 0.340). The same results were obtained for freedom from AF within the first year post-ablation (*p* = 0.161) or from 1 year until the end of the study (*p* = 0.054) (Figures 2B,C). Remarkably, an intriguing crossover of the recurrence curves was observed after about 1,500 days during the full study follow-up. AF recurrences in patients with paroxysmal AF or persistent AF showed no significant differences between both the intervention and control groups during the study follow-up (paroxysmal: 66.5% vs. 70.5% respectively; persistent: 32.5% vs. 28.9% respectively).

### Factors associated with AF recurrences

Table 2, Supplementary Tables S5 and S6 shows the results of the multivariate logistic regression model from PVI until the end of the study, from PVI within 1-year post-ablation and from 1-year PVI until the end of the study, respectively. In general, all three logistic regression models report pacemaker and redo-ablation as having a statistically significant association with AF recurrence. When looking at either the whole study period or from 1-year post-PVI until the end of the study, the following variables are also statistically significant associated with AF recurrence in addition to the previous mentioned variables: gender, hypertension, the treatment with antiarrhythmic drugs at month 3. For the whole study period, also sleep apnea, the ejection fraction and having CVA after PVI, have a statistically significant association with the outcome. However, logistic regression analysis indicated that participating in the CR program was not a significant predictor of AF recurrence during the whole study period [OR: 0.876; 95% CI (0.671–1.142); *p* = 0.328]. The Nagelkerke *R*^2^ of 0.342 indicates that the model has a moderate fit, suggesting that the included predictors improve the ability to distinguish between patients with and without AF recurrence compared to a model with no predictors. From 1-year post-PVI until the end of the study, the logistic regression analysis indicated that patients who participated in the CR program had a 32.2% lower odds of experiencing AF recurrences [OR: 0.677; 95% CI (0.514–0.891); *p* = 0.005]. There was an additional different factor with significant association from 1 year post-PVI until the end of the study, i.e., having a cardiac surgery.

**Table 2:** The multivariate logistic regression model with AF recurrence as outcome from PVI until the end of the study in the total population.

Independent variables	Multivariate analysis		
	OR	95% CI	*p*-value
Age	1.007	0.994–1.021	0.291
Female gender	0.777	0.589–1.024	**0** **.** **073**
Hypertension before PVI	1.557	1.194–2.031	**0** **.** **001**
Diabetes before PVI	0.981	0.638–1.510	0.932
Sleep apnea before PVI	1.724	1.093–2.719	**0** **.** **019**
COPD/asthma before PVI	0.979	0.627–1.528	0.926
Heart failure before PVI	1.027	0.710–1.485	0.889
Coronary artery disease before PVI	0.880	0.616–1.256	0.480
Severe heart valve before PVI	1.278	0.543–3.008	0.575
Following cardiac rehabilitation	0.876	0.671–1.142	0.328
CVA after PVI	3.757	1.163–12.136	**0** **.** **027**
Pacemaker before PVI	1.900	1.207–2.992	**0** **.** **006**
Ejection fraction before PVI	1.018	1.002–1.034	**0** **.** **032**
Antiarrhythmic use at month 3 after PVI	1.460	1.128–1.889	**0** **.** **004**
Type of AF ablation			0.956*
Radiofrequency (reference category)			
Laser	1.621	0.065–40.571	0.769**
Electroporation	1.606	0.064–40.290	0.773**
Redo ablation	18.302	12.954–25.860	**<0** **.** **001**

AF, atrial fibrillation; CI, confidence interval; COPD, chronic obstructive pulmonary disease; CVA, cerebrovascular accident; OR, odds ratio; PVI, pulmonary vein isolation.

* *p*-value indicates overall significance.

** *p*-values indicate significance for the categories.

Bold values are the parameters with a significant *p* value.

### Total number of recurrences within 1 year post-PVI

**Figure 3:**
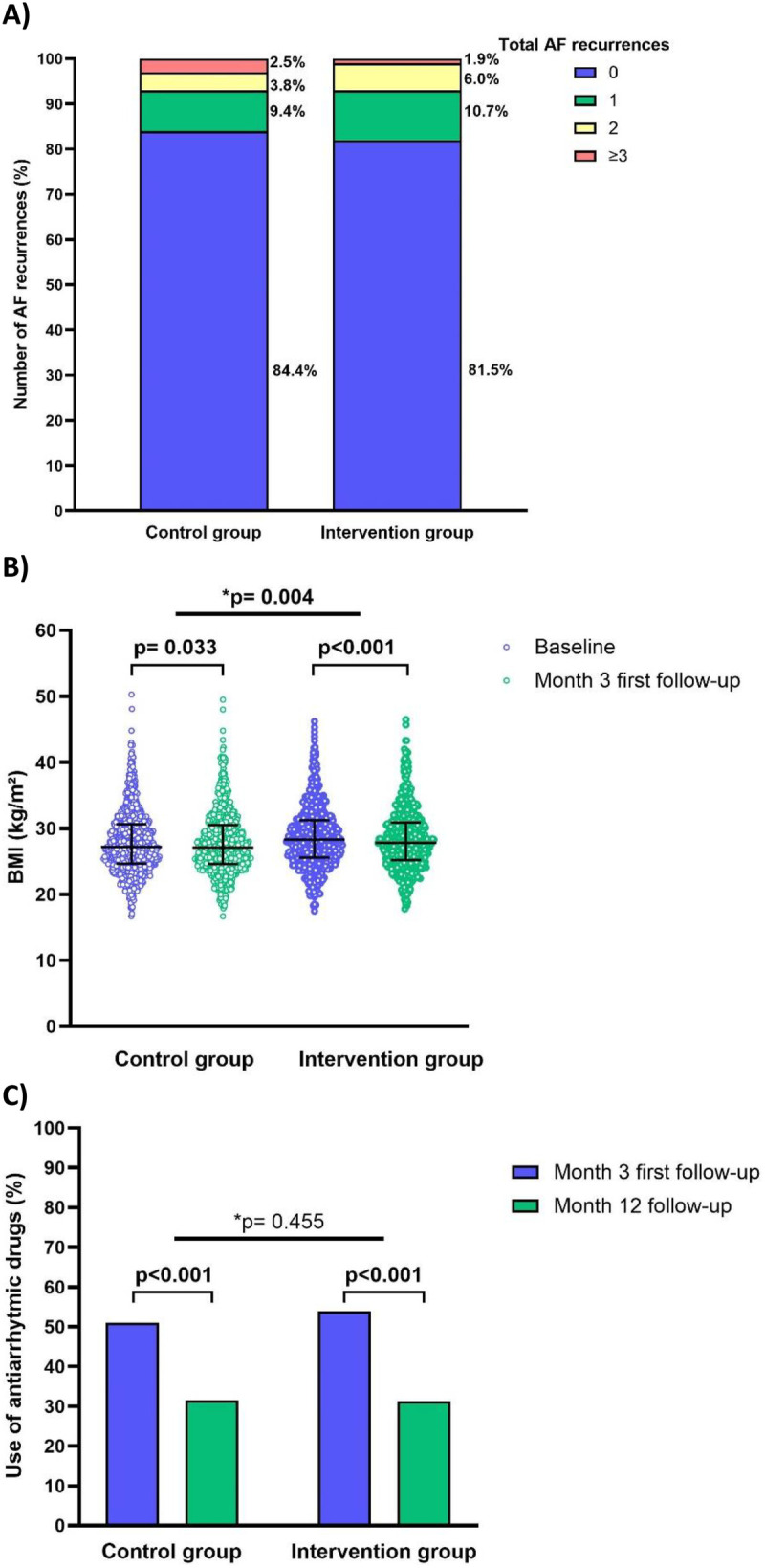
**(A)** Total number of AF recurrences within 1 year post-PVI ablation (with 3 months initial blanking period) in the control and intervention group (*n* = 588). AF, atrial fibrillation. **(B)** Changes in the BMI since PVI ablation in the control and intervention group. Data are represented as scatter dot plots with median and interquartile range. For the statistics, the Wilcoxon signed-rank test and the Linear Mixed Model test was performed. The *p* value refers to within-group differences (baseline to month three first follow-up), *p value refers to between-group differences over time (group-time interaction). BMI, body mass index. **(C)** The use of antiarrhythmic drugs since PVI ablation in the control and intervention group. The data are represented as column bar graphs. For the statistics, the McNemar test and the Generalized Linear Mixed Model test was performed. Blue colour represents the follow-up at 3-month post-ablation and green colour represents the follow-up at 1-year post-ablation. The *p* value refers to within-group differences (baseline to follow-up), *p value refers to between-group differences over time (group-time interaction). *P*-value <0.05 was considered as statistically significant.

Figure 3A shows the total number of AF recurrences within 1 year post-PVI. The results demonstrate that 84.4% of the control patients experienced no AF recurrence, while 9.4% had one recurrence, 3.8% had two recurrences, and 2.5% had three or more recurrences. Similar results (*p* = 0.280) were obtained in the intervention group, of which 81.5% of the patients had no recurrences, while 10.7% experienced one recurrence, 6.0% two recurrences, and 1.9% experienced three or more recurrences. In case of a recurrence, the mean number of total AF recurrences was also comparable in both groups (control group: 2.2 ± 2.4 and intervention group: 1.6 ± 0.8; *p* = 0.423).

### Evolution of patients' BMI and the continuation of antiarrhythmic drug intake

Figures 3B,C show the changes in patients’ BMI and the use of anti-arrhythmic agents since the PVI ablation. The intervention group had a significantly lower BMI (28.3 vs. 27.8 kg/m^2^; *p* < 0.001) at 3-month follow-up compared to baseline, while the control group had a significantly higher BMI (27.2 vs. 27.8 kg/m^2^; *p* = 0.033) at 3-month follow-up compared to baseline. The decrease in BMI was also significantly different in both groups (*p* = 0.004). At 1-year follow-up, the control group had 20.3% fewer patients (51.8% vs. 31.5%; *p* < 0.001) still taking antiarrhythmic medications compared to the 3-month follow-up. The decrease was 22.6% in the intervention group (53.9% vs. 31.3%; *p* < 0.001). The between-group differences over time was comparable (*p* = 0.455).

### Rehabilitation parameters

**Figure 4:**
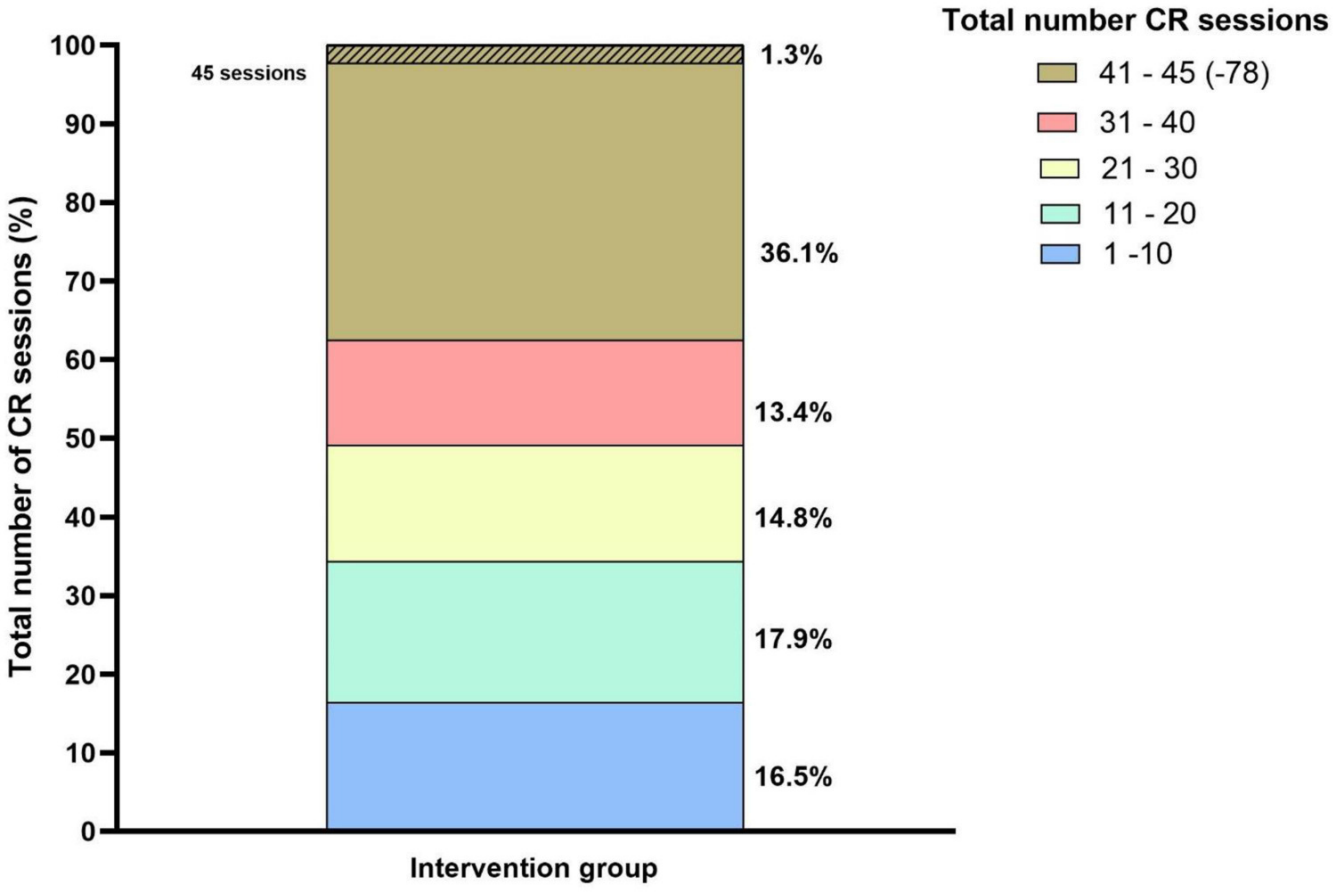
Total number of CR sessions of the intervention group after PVI ablation. Blue colour represents 1–10 sessions, green colour 11–20 sessions, yellow 21–30 sessions, red colour 31–40 sessions, brown 41–78 sessions, of which the shaded part represents more than 45 sessions. CR, cardiac rehabilitation.

The total number of CR sessions completed by the intervention group is shown in Figure 4. Unfortunately, in half (*n* = 297; 50.5%) of the CR patients, it is unknown how many CR sessions they participated in. The median number of completed CR sessions is 32 (15–44), and from the known data, 36.1% of the patients completed between 41 and 45 sessions. VO_2_ max and maximal power were significantly increased at the end of the CR program (*p* < 0.001): VO_2_ max increased from a median of 19.9 (15.7–24.9) mL/kg/min to 22.3 (18.4–26.6) mL/kg/min, and maximal power from a median of 135 (96.3–186) to 158 (109–210) Watts.

## Discussion

This retrospective study examined the impact of a CR program on AF recurrences in patients after a first PVI ablation between 2007 and 2020. Currently, physical inactivity is a challenging factor in keeping a healthy lifestyle. Although CR programs have shown clinical benefits in various cardiovascular conditions, evidence is lacking in patients with AF (13). However, in this study, the CR program was not associated with a lower AF recurrence rate in post-PVI patients. Nevertheless, physical activity from the CR program may have long-term benefits based on the proven weight loss and increase in VO_2_ max, as may also be suggested by an intriguing crossing of the AF recurrence curves from 1 year post-PVI.

### Impact of CR program on AF, physical capacity, and BMI

This study shows that patients who opted to participate or not in an additional CR program after PVI experienced comparable AF recurrences (18.9% vs. 15.9%, respectively) within 1 year post-ablation. This was not in line with a previous study, in which the AF recurrence rate was 25.9% for the rehabilitation group and 31.4% for the control group (*p* = 0.520) (14). A systematic review by Kurasawa et al. showed that the decreased AF reduction rate in the CR group was not statistically significant (*p* = 0.150) (15). However, subgroup analyses within BMI categories indicated that patients with a BMI less than 30 kg/m^2^ experienced a statistically significant reduction in AF, while those with a BMI greater than 30 kg/m^2^ did not show any significant reduction (15). This means that the patient characteristics of the study population could also influence the AF recurrence rate. In addition, an important aspect is the frequency of heart rhythm monitoring. In this retrospective study, no continuous monitoring was performed. Interestingly, the survival analyses from PVI until the end of the study follow-up showed a crossover of curves (at 1,500 days), which could be explained by a higher probability of AF detection in the intervention group. Logistic regression showed that “participating in the CR program” was a significant predictor (*p* = 0.005) of AF non-recurrence from 1 year until the end of the study. This could be an indication that physical activity from the CR program may have long-term advantages on AF recurrence from 1 year post-PVI, based on the proven weight loss and increase in VO_2_ max.

The early detection of AF recurrences in the intervention group could be explained by the closer follow-up of the CR patients, i.e., these patients were seen more often by a healthcare provider, leading to a possible earlier detection of AF recurrence. Nevertheless, the CR program shows a tendency towards a positive impact, when we look at the results of the logistic regression analysis from 1 year post-PVI until the end of the follow-up. In addition, the multivariate model yielded Nagelkerke *R*² values of 0.342 (from PVI until the end of the study) and 0.241 (from 1 year until the end of the study), indicating the relative improvement in model fit for predicting AF recurrence compared to a model including only intercept.

Moreover, this study showed a significant improvement in VO_2_ max (19.9–22.3 mL/kg/min) and maximal power (135.0–158.0 Watt) in the intervention group at the end of the CR program (*p* < 0.001). Similar results were obtained in a randomised trial with 61 patients, of which 31 patients followed a CR program after their ablation (VO_2_ max: 17.8–19.8 mL/kg/min and Power max: 105.0–124.0 Watt) (16). Nevertheless, no difference in AF recurrence was found between both groups, as seen in this retrospective study. According to the authors of this randomised trial, exercise training improved the cardiac function in patients with persistent AF after ablation. There are many ways via which exercise training and multidisciplinary rehabilitation may improve outcomes after AF ablation, but it is clear that more research is needed to define and optimise those mechanisms (16–18). In addition, a meta-analysis of Buckley et al. also showed an improvement in VO_2_ max (19).

The results showed that patients in the intervention group experienced an absolute decrease of 0.5 kg/m^2^ in their BMI, from 28.3 to 27.8 kg/m^2^, while the control group had an absolute increase of 0.6 kg/m^2^ in BMI at 3-months post-ablation. This difference can be explained by the positive effect of the CR program on BMI, the fact that patients had the opportunity to participate in information sessions, such as on weight control and healthy diet, and obviously the personal coaching from the dietitian. Another comparative study in patients with peripheral artery disease observed about a decrease of about 3 kg/m^2^ in BMI after the CR program (20). Interestingly, insights from the EU-PORIA Registry showed that a one-unit rise in BMI was linked to a 4.2% higher risk of recurrence for AF/atrial tachyarrhythmia (21).

### Study limitations

Some patients (*n* = 127) were excluded from the study due to missing data as they were partially followed up in another hospital. Obviously, this study contains the intrinsic limitation of a single centre, non-randomised retrospective trial. It is challenging to gather all AF recurrences because it is only possible to consider those recorded in the medical files (e.g., confirmed ECG, planned cardioversion, or redo ablation), leading possibly to underestimation of the AF recurrence rate. However, a 24-hour Holter was not part of the general follow-up at 3 and 12 months post-PVI for all patients, which was an important aspect for this study to collect the AF recurrences at these timepoints. On the other hand, no information on AF recurrence was collected during the blanking period, as it may have had lesser clinical value. However, this data could be useful for declaring the crossover. The retrospective nature leads to unequal distribution of both groups (control group: *n* = 1,177 and intervention group: *n* = 588) since it was up to the patient to decide whether to follow the CR program. There may be implicit bias related to certain patient characteristics, which could be associated with both unwillingness to follow a CR program and an increased risk of AF recurrences. Important potential confounders, such as physical activity level, diet, adherence to preventative measures, etc…, were not collected in this study. Therefore, they could not be included in the statistical analysis. As a result, confounding may be present and should be considered when interpreting the findings of this study. Moreover, a sensitivity analysis was conducted in which parametric survival models were used to compare the survival distributions of the control group and CR group. The model was adjusted for important clinical covariates, which were also used in the logistic regression model. Several distributions were used, such as a Weibull lognormal, loglogistic and gamma distribution in order to improve the fit of the model. However, an adequate fit of the model could not be obtained, but the results were consistent among the tried distributions, that there was no statistical difference between AF-free survival time between patients following standard of care and patients that had rehabilitation. On the other hand, to identify potential risk factors for AF recurrence, we used a stepwise variable selection procedure while forcing clinically important covariates into all models. Although stepwise selection is commonly used in exploratory analyses, it has limitations, including the possibility that different samples may yield different selected predictors. Confirmation of the findings reported here will require a prospective randomised controlled trial. Another limitation is that not all CR patients completed the 45 sessions of their CR program and stopped earlier. This means it is difficult to get a clear picture of how their physical exercise affects their recurrence because of a different total number of CR sessions. Lastly, the outcomes could have been further enhanced with a more extended CR program since Belgium’s reimbursement policy, like in many other countries, limits the number of supervised CR sessions.

### Future perspectives

As mentioned in the ESC guidelines for the management of AF, managing comorbidities (including improving physical activity) is an important factor in the overall care of AF patients. Therefore, it is essential to know whether CR programs impact on AF recurrence. This will necessitate more prospective trials with continuous heart rhythm monitoring after PVI ablation.

## Conclusion

This retrospective study did not show that post-PVI patients who participated in a structured CR program had a lower AF recurrence rate. Nevertheless, different predictors for AF recurrence were determined, and physical activity from the CR program may have long-term benefits from 1 year post-PVI, based on the proven weight loss and increase in VO_2_ max. Furthermore, the functional exercise capacity of the CR patients significantly improved during the CR program. More prospective studies with continuous heart rhythm monitoring are necessary to record all the AF recurrences and better assess the association between the CR program and AF recurrences.

## Data Availability

The data underlying this article will be shared on reasonable request to the corresponding author.
