# Supplementary material for: The impact of cardiac rehabilitation on atrial fibrillation recurrence after pulmonary vein isolation: results of a large retrospective study
Source: Front Cardiovasc Med. 2026 Jan 27;12:1717749. doi: 10.3389/fcvm.2025.1717749 (PMC12887724; doi:10.3389/fcvm.2025.1717749)
Supplement: Supplementary file 1 [file Table1.docx]

Supplementary file

**Supplementary table 1**

**All collected parameters during the study follow-up.**

| Group | All collected parameters |
| --- | --- |
| General | Age, gender, weight, height, and BMI. |
| Risk factors and comorbidities | Family history, hypertension, diabetes, hyperlipidemia, smoking, sleep apnea, coronary artery disease, heart failure, obstructive pulmonary disease, TIA/CVA, cardiac surgery, pacemaker, an artificial valve, a severe heart valve disease, and terminal renal failure. |
| Echocardiographic parameters | Ejection fraction, enlargement of left atrium, and mitral valve regurgitation. |
| Laboratory parameters | Renal function, CRP, fasting glycaemia, HbA1c, hyperthyroidism, and hypothyroidism. |
| AF-related parameters | Type of AF, type of AF ablation, duration of AF before the first ablation, AF recurrence between three-months and one-year, date of the first AF recurrence between three-months and one-year, total number of AF recurrences between three-months and one-year, AF recurrence after one-year, and date of first AF recurrence after one-year until end of the study. |
| Medication | Antiarrhythmics and beta-blockers. |
| Rehabilitation group | The total number of rehabilitation sessions, VO_2_ max and power max. |
| Other parameters | Complicated ablation requiring an extended hospital stay (e.g. tamponade,…), and death. |

BMI: Body mass index; TIA: Transient ischemic attack; CVA: Cerebrovascular accident, CRP: C-reactive protein,

HbA1c: Haemoglobin A1c; AF: Atrial fibrillation; VO2: maximal oxygen consumption.

**Supplementary Table 2**

**Variables used to investigate the association with AF recurrence.**

| Variables used to investigate the association with AF recurrence |
| --- |
| Family cardiac history |
| Hyperlipidemia |
| Smoking history |
| CVA |
| Cardiac surgery |
| Pacemaker |
| Artificial valve |
| Ejection fraction |
| Enlargement of the left atrium |
| Type of AF |
| Type of ablation |
| Hyperthyroidism |
| Hypothyroidism |
| Antiarrhythmics at month 3 post-PVI |
| Redo ablation |

AF: Atrial fibrillation, CVA: Cerebrovascular accident, PVI: Pulmonary vein isolation.

**Supplementary table 3**

**The multivariate logistic regression model with AF recurrence as outcome**

**within one year post-PVI in the total population.**

|  | Multivariate analysis | | |
| --- | --- | --- | --- |
| Independent variables | OR | 95% CI | **P-Value** |
| Age | 1.014 | 0.998-1.029 | 0.082 |
| Gender | 0.906 | 0.662-1.240 | 0.537 |
| Hypertension before PVI | 0.928 | 0.692-1.244 | 0.619 |
| Diabetes before PVI | 1.446 | 0.896-2.332 | 0.131 |
| Sleep apnoea before PVI | 1.265 | 0.764-2.094 | 0.361 |
| COPD/asthma before PVI | 0.610 | 0.346-1.073 | 0.086 |
| Heart failure before PVI | 0.715 | 0.464-1.103 | 0.129 |
| Coronary artery disease before PVI | 1.060 | 0.713-1.575 | 0.774 |
| Severe heart valve before PVI | 0.425 | 0.126-1.428 | 0.166 |
| Rehabilitation | 1.324 | 0.988-1.775 | 0.060 |
| Pacemaker before PVI | 1.923 | 1.146-3.227 | **0.013** |
| AF type   - Paroxysmal (reference category) - Persistent - Permanent | 1.446  4.044 | 1.062-1.968  1.056-15.483 | **0.012**  **0.019**  **0.041** |
| Redo ablation | 10.715 | 8.020-14.316 | **<0.001** |

AF: Atrial fibrillation; CI: Confidence interval; COPD: Chronic obstructive pulmonary disease;

OR: Odds ratio; PVI: Pulmonary vein isolation.

**Supplementary table 4**

**The multivariate logistic regression model with AF recurrence as outcome**

**from one year post-PVI until end of the study in the total population.**

|  | Multivariate analysis | | |
| --- | --- | --- | --- |
| Independent variables | OR | 95% CI | **P-Value** |
| Age | 0.996 | 0.982-1.010 | 0.553 |
| Gender | 0.639 | 0.485-0.843 | **0.002** |
| Hypertension before PVI | 1.715 | 1.316-2.235 | **<0.001** |
| Diabetes before PVI | 0.674 | 0.424-1.070 | 0.094 |
| Sleep apnoea before PVI | 1.157 | 0.727-1.842 | 0.538 |
| COPD/asthma before PVI | 1.367 | 0.899-2.078 | 0.144 |
| Heart failure before PVI | 0.862 | 0.599-1.240 | 0.423 |
| Coronary artery disease before PVI | 1.778 | 0.544-1.142 | 0.208 |
| Severe heart valve before PVI | 1.157 | 0.499-2.686 | 0.734 |
| Rehabilitation | 0.658 | 0.501-0.865 | **0.003** |
| Pacemaker before PVI | 1.647 | 1.017-2.669 | **0.043** |
| Cardiac surgery before PVI | 1.757 | 1.087-2.840 | **0.022** |
| AF type   - Paroxysmal (reference category) - Persistent - Permanent | 1.812  4.923 | 1.376-2.387  1.332-18.194 | **<0.001**  **<0.001**  **0.017** |
| Redo ablation | 6.695 | 5.082-8.822 | **<0.001** |
| Antiarrhythmic use at month 3 after PVI | 1.449 | 1.113-1.886 | **0.006** |

AF: Atrial fibrillation; CI: Confidence interval; COPD: Chronic obstructive pulmonary disease;

OR: Odds ratio; PVI: Pulmonary vein isolation.
